# Knee septic arthritis caused by α-hemolytic Streptococcus in a patient with a recent history of knee arthroscopy: a case report

**DOI:** 10.1186/s12879-019-4556-4

**Published:** 2019-10-24

**Authors:** Giovanni Balato, Tiziana Ascione, Paolino Iorio, Cristiano De Franco, Vincenzo De Matteo, Alessio D’Addona, Nicola Tammaro, Achille Pellegrino

**Affiliations:** 1 Unit of Orthopaedic Surgery, Department of Public Health, School of Medicine, Federico II University, Naples, Italy; 2 Department of Infectious Diseases, D. Cotugno Hospital, AORN Dei Colli, Naples, Italy; 3 Department of Orthopedics, Traumatology, Plastic-Reconstructive and Rehabilitation, School of Medicine, Luigi Vanvitelli University, Naples, Italy; 4 Unit of Orthopedics and Traumatology, S.G. Moscati Hospital, CE, Aversa, Italy

**Keywords:** Knee, Septic arthritis, Arthroscopic partial meniscectomy, *Streptococcus spp*

## Abstract

**Background:**

Arthroscopic partial meniscectomy is a common procedure in orthopedic practice. Infections are uncommon complications of this procedure with an incidence rate of 0,01% - 3,4%. *Staphylococcus spp* are the predominant causative agents in such cases. We present a case of knee septic arthritis caused by *α-hemolytic Streptococcus*.

**Case presentation:**

A 22-year-old woman diagnosed with obesity (body mass index [BMI] 35 kg/m^2^) but with no other major comorbidities underwent an arthroscopic selective meniscectomy with administration of intravenous cefazolin for antibiotic prophylaxis. After an uneventful period of 2 months, the patient returned with pain, fever and a discharging sinus at the site of anterolateral arthroscopic portal. Blood tests and magnetic resonance imaging revealed osteomyelitis involving the tibial plate. Cultures of synovial fluid obtained from the knee and a pharyngeal swab yielded *α-hemolytic Streptococcus*. Five days later, the patient underwent arthroscopic debridement with partial synovectomy. Intraoperative specimens yielded *α-hemolytic Streptococcus*. The patient received intravenous piperacillin/tazobactam, followed by an associative regimen of amoxicillin and clindamycin with clinical, laboratory and instrumental evidence of symptom resolution.

**Conclusion:**

The incidence of knee septic arthritis after arthroscopic partial meniscectomy is 0.01–3.4%. This infection is usually caused by *Staphylococcus spp.* and in rare cases by commensal bacteria, such as α-hemolytic streptococci, secondary to transient bacteremia. Screening of the colonized area is important to prevent possible transient bacteremia. Diagnosis is based on isolation of the causative organisms from synovial fluid cultures, and treatment comprises arthroscopic debridement with individualized systemic antibiotic therapy based on the results of an antibiogram.

## Background

Arthroscopic partial meniscectomy is one of the most common procedures in orthopedic practice. It is a reliable and cost-effective technique associated with good outcomes, low complication rates, and rapid recovery of function of the operated knee. Arthroscopy is performed for meniscal tears that cause pain and functional limitations. Outcomes of arthroscopic partial meniscectomy are affected by age, sex, type and location of meniscal tears, concomitant knee instability, and degenerative or traumatic articular lesions [1, 2].

Infections are uncommon complications of arthroscopy with an incidence rate of 0.01–3.4%. Postsurgical septic arthritis is defined based on the following features: i) clinical presentation of fever, local pain, edema, erythema or tenderness; ii) laboratory investigations showing leukocytosis with elevated erythrocyte sedimentation rate (ESR), and C-reactive protein (CRP); iii) synovial fluid examination showing a white blood cell (WBC) count > 2.5 × 10^4^/μL or positive synovial fluid culture results [3].

Common risk factors for post-arthroscopic septic arthritis include open arthrotomy, procedure involving implants such as anterior cruciate ligament (ACL) allografts (which constitute foreign bodies), age > 50 years and tourniquet time > 60 min [4–6]. *Staphylococcus aureus* and Coagulase Negative *Staphylococci* are the most common causative agents, while *Streptococci* are involved with a lower frequency. The incidence of infections caused by *α-hemolytic Streptotoccus* spp. is not described in the available literature, and limited anecdotal data are available from case reports [7, 8]. *Streptococcus spp* cause conjunctivitis, meningitis, bacterial pneumonia endocarditis, erysipelas and necrotizing fasciitis (“flesh-eating” bacterial infections), in addition to streptococcal pharyngitis [9].

Classification of *Streptococcus spp* is based on the hemolytic properties of these bacteria. Alpha-hemolytic species cause oxidization of iron in hemoglobin molecules within red blood cells, producing green-colored colonies on blood agar. *Streptococcus pneumoniae* and a group of oral streptococci (*S. viridans*) tipically cause alpha hemolysis [10].

We report a rare case of 22-year-old woman with obesity who developed septic arthritis secondary to α-hemolytic streptococcal infection after simple arthroscopy.

## Case presentation

Written informed consent for publication of their clinical details and/or clinical images was obtained from the patient. A copy of the consent form is available for review by the Editor of this journal.

A 22-years-old woman with obesity (BMI 35 kg/m^2^) with no major comorbidities underwent arthroscopic selective lateral meniscectomy in March 2017. She received intravenously administered cefazolin (2 g) for antibiotic prophylaxis an hour before the skin incision, based on the protocol followed by our hospital and guidelines reported in the available literature [11]. Her postoperative recovery was uneventful until 2 months after the operation.

**Fig. 1:**
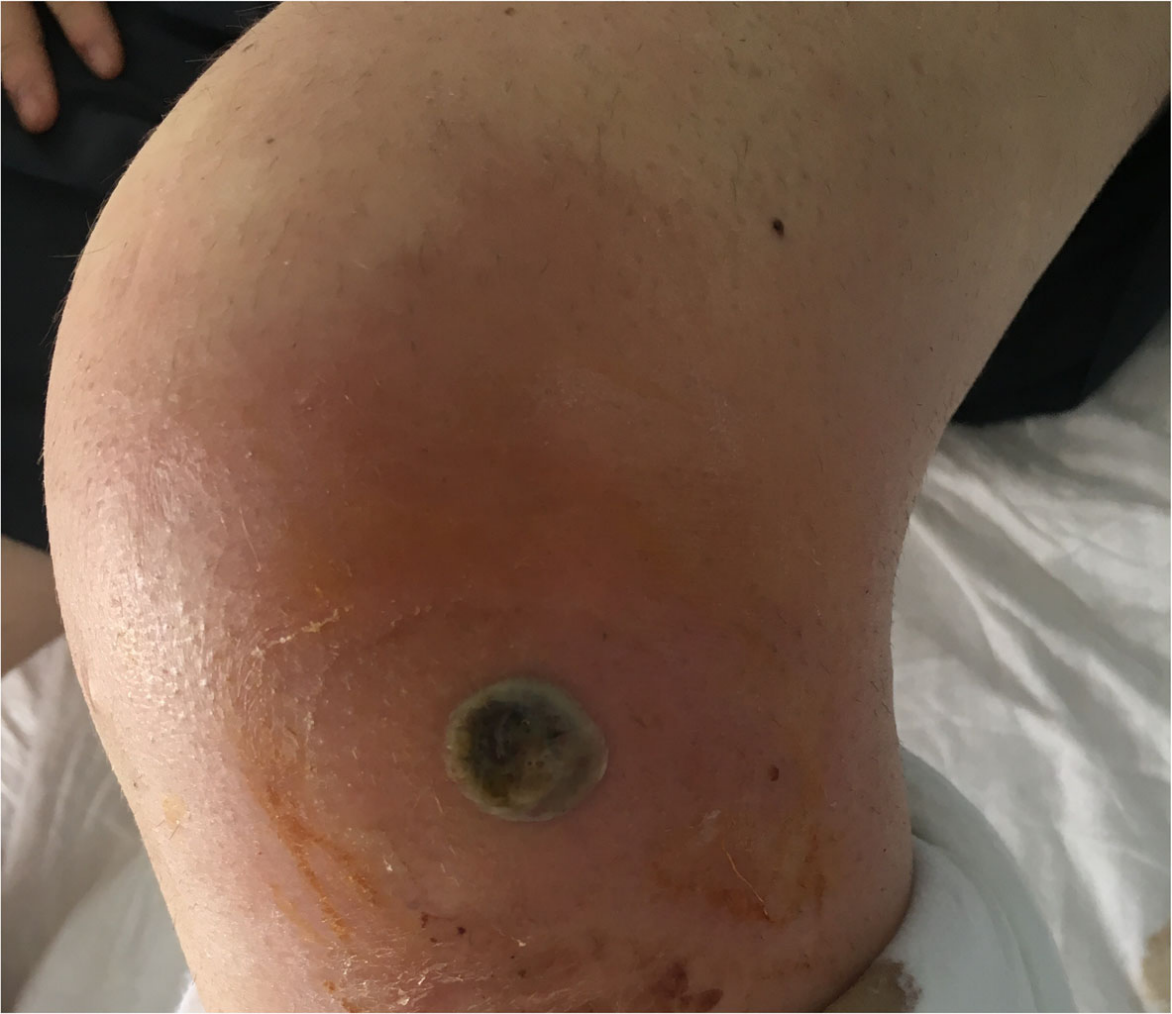
Two months after the surgical procedure, the patient returned to control. In the post-operative period a fistula was observed on the site of the antero-lateral arthroscopic portal

**Fig. 2:**
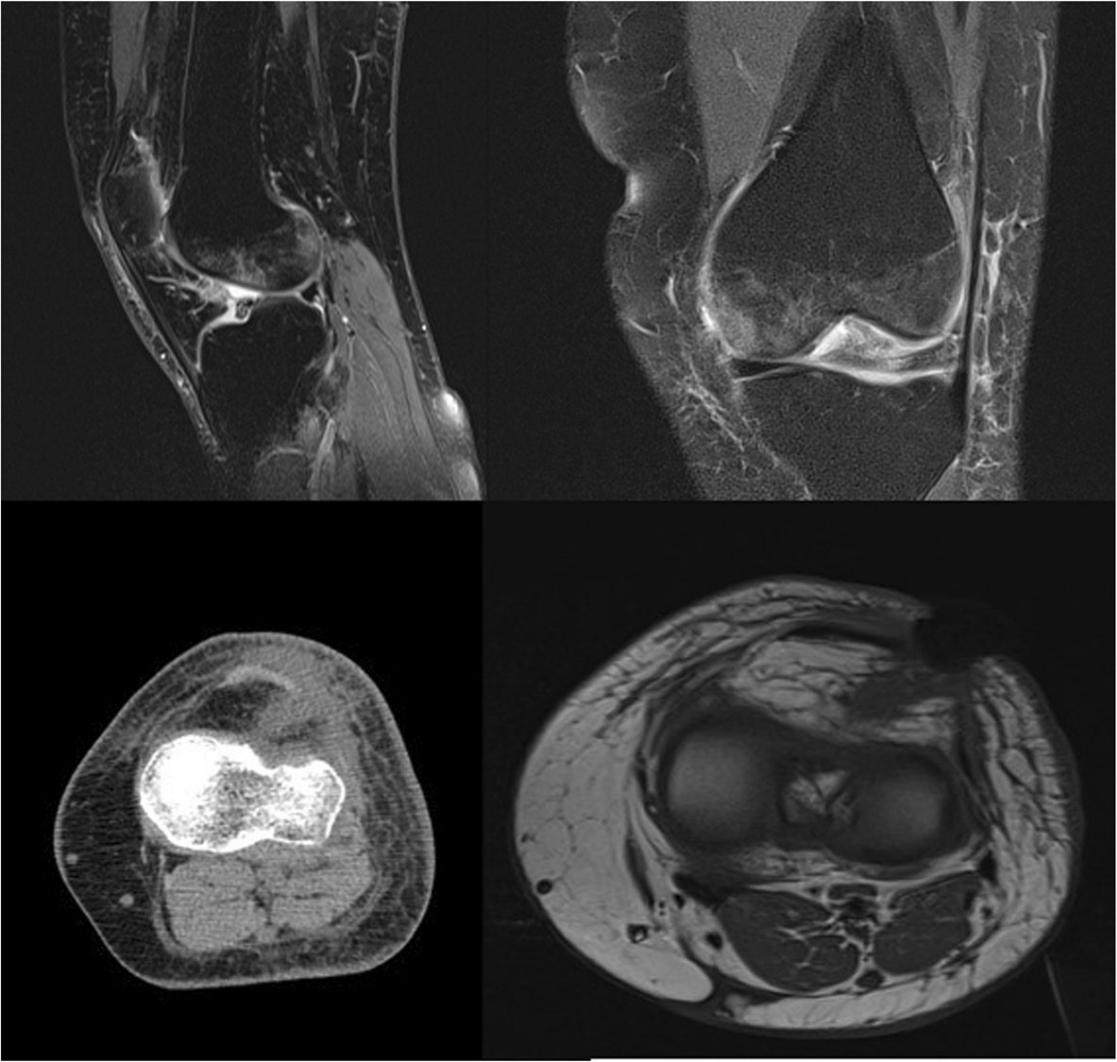
The MRI study of the knee shows signs of septic arthritis, with bone edema and abscesses around surgical site and next to Hoffa. The presence of imbibition and swelling of the periarticular tissues (cutaneous and subcutaneous) can be observed

The patient was readmitted to the orthopedic department in May 2017 (8 weeks postoperatively) with pain, reduced mobility of the operated knee, and spiking fever > 38.5 °C. Physical examination on admission revealed a painful and edematous operated knee with a discharging sinus observed at the site of the anterolateral arthroscopic portal (Fig. 1). Laboratory examination revealed an elevated ESR (55 mm/h) and serum CPR (40 mg/L). Blood and urine cultures were sterile. Magnetic resonance imaging (MRI) of the operated knee revealed evidence of septic arthritis with bone edema and abscesses surrounding surgical site (Fig. 2). Knee joint aspiration was performed for laboratory and microbiological investigations, which showed an elevated WBC count (54.000 cells/uL) with elevated levels of polymorphonuclear cells (93%). Synovial fluid was cultured and yielded *α-hemolytic Streptococcus*.

Pharyngeal swabs obtained on 2 consecutive days yielded the same α*-hemolytic Streptococcus,* which were isolated earlier from aerobic cultures. It should be remembered that α-hemolytic streptococci may be members of the normal oropharyngeal flora [12]. Transthoracic echocardiography was performed to rule out possible bacterial endocarditis leading to haematogenous spread of infection to knee joint. However, this examination revealed no abnormalities.

Five days after the patient was readmitted to the orthopaedic department, arthroscopic debridement with partial synovectomy was performed, and teicoplanin (8 mg/kg body weight) was administered daily while awaiting culture results to ensure adequate coverage for methicillin-resistant *S. aureus*, based on our hospital protocol. Cultures of intraoperative tissue samples confirmed the growth of α*-hemolytic Streptococcus* susceptible to penicillin G, and the patient was administered piperacillin/tazobactam (13.5 g/day) with rapid resolution of local signs and symptoms and improved serum CRP and ESR levels. Intravenous antibiotic therapy was continued for 2 weeks without significant adverse effects, based on the outpatient parenteral antibiotic therapy (OPAT) model. Two weeks after arthroscopic debridement, intravenous antibiotic therapy was switched to oral therapy with amoxicillin (1 g thrice daily) and clindamycin (150 mg twice a daily), for 8 weeks.

**Fig. 3:**
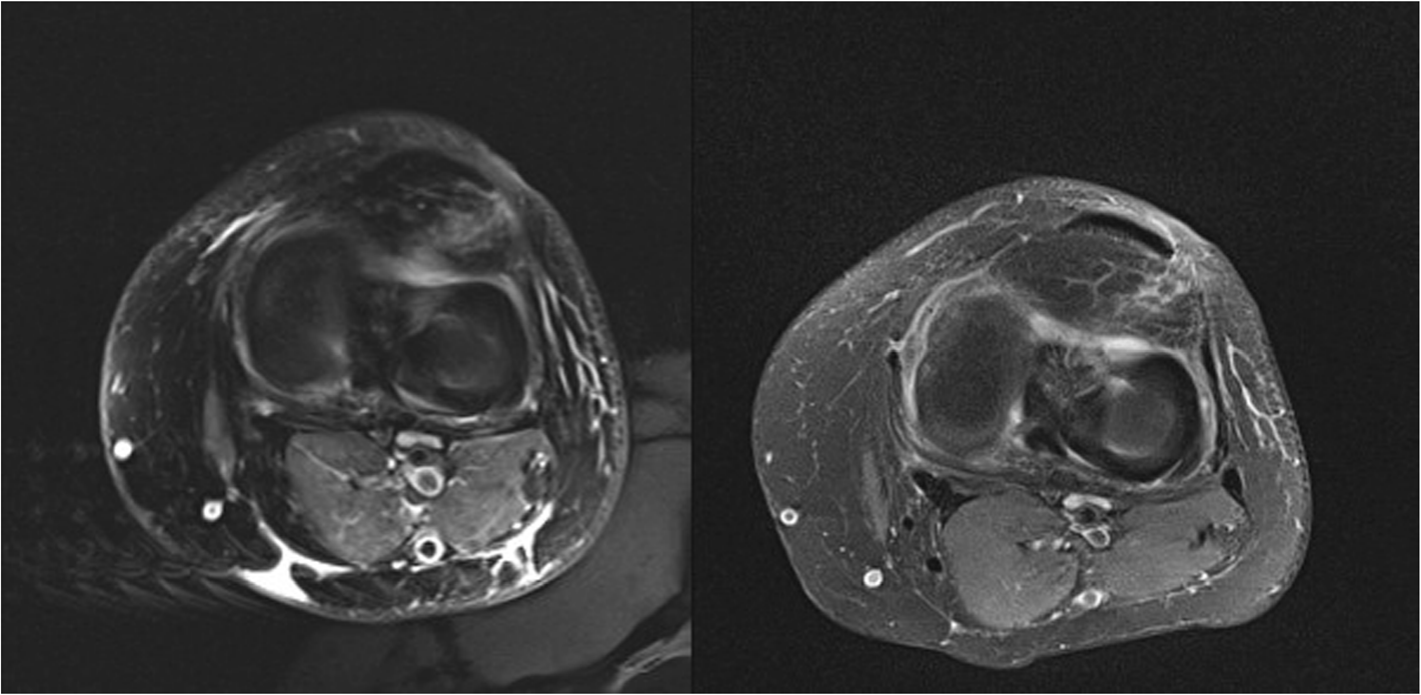
MRI after 30 (on the left) and 60 (on the right) days: the images show a reduction of imbibition and swelling of the periarticular tissues; besides the abscesses around the surgical site is not more detectable

In July 2017 (8 weeks after debridement), ERS and CRP levels returned to the normal range with complete resolution of local signs of infection, and MRI revealed complete healing (Fig. 3).

In October 2017 (24 weeks after debridement), the patient did not show any evidence of infection. Functional evaluation revealed good results with complete range of motion (ROM), and the patient scored 100/100 points each on Lysholm Knee Scoring Scale as well as Knee injury and Osteoarthritis Outcome Score (KOOS).

## Discussion and conclusions

Knee arthroscopy is one of the most common procedures performed by orthopedic practice. According to the American Orthopaedic Surgery database from 2003 to 2009, the overall complication associated with arthroscopic knee procedures was approximately 4.7%.

Studies have shown that the overall complication rate associated with partial meniscectomies was approximately 2.8% and that infections constitute the most common complication (0.01–3.4%) [13]. The incidence rate of infections requiring surgery after knee arthroscopy is approximately 0.15%, with higher rates observed in men and in patients undergoing arthroscopies that require placement of implants [14].

Post-arthroscopic knee infections are more common in patients with obesity, smokers, patients undergoing complex procedures, men, patients with diabetes, and in procedures with time > 60 min [15–18]. Surgical site infections (SSIs) are usually caused by commensals. Reports in the literature show that patient with *S. aureus* colonization in their nasal mucosa or skin are at a higher risk of SSIs. Several studies have reported an association between bacteria isolated from patients with SSI and their commensal flora [19–25]. Transient bacteremia from colonized site is associated with the theoretical risk of hematogenous spread of bacteria and seeding at the surgical site. Notably, α-hemolytic streptococci are a large group of bacteria that are members of the oral commensal flora; however, in some cases, they can colonize distant organs (for example, in patients with diseases such as endocarditis [*S. viridans*]) [25]. To our knowledge, no report has described post-arthroscopic septic arthritis of the knee caused by α-hemolytc streptococci, although staphylococcal joint infections are widely reported in the available literature [26–28]. We conclude that septic arthritis of the knee could be secondary to transient α-hemolytic streptococcal bacteremia following seeding of oral flora at a site of recent surgery where necrotic tissue favors bacterial growth. Tobacco use, obesity, diabetes, age > 50 years, and high-complexity procedures serve as risk factors for SSIs [28–31]. Diagnosis is based on culture examination of aspirated synovial fluid. Urgent therapeutic measures include copious arthroscopic irrigation and lavage, synovectomy, and concomitant administration of 2 effective antibiotics (based on the antibiogram) for at least 6 weeks [32]. Johns et al. [33] reported that outcomes of arthroscopic procedures were comparable with those of open surgery. Appropriate antibiotic therapy achieves effective eradication of infection in 85% of cases without any adverse effect on final function of the affected knee [34, 35].

Post-arthroscopic knee septic arthritis is a complication associated with significant morbidity. We report a rare case of post-arthroscopic septic knee arthritis in a young woman with obesity without any other risk factors predisposing to knee infection, including bacterial endocarditis that is known to cause hematogenous spread of infection to the knee. Septic arthritis of the knee is a serious complication of arthroscopy, which is usually caused by the *Staphylococci spp.* and by uncommon pathogenic bacteria in rare cases. We isolated *α-hemolytic Streptococcus* following simple arthroscopy in our patient in whom the infection could be attributed to transient streptococcal bacteremia originating from the commensal oral flora of the patient. Therefore, we recommend screening the colonized area even in patients undergoing simple surgical procedures. Diagnosis in based on culture examination of aspirated synovial fluid. Arthroscopic debridement with administration of systemic antibiotics led to resolution of the infection in our patient.

## Data Availability

No datasets were generated or analysed during the current study. Data sharing is available from corresponding author on reasonable question.

## Abbreviations

ACL: Anterior cruciate ligament
BMI: Body mass index
CRP: C-reactive protein
ESR: Erythrocyte sedimentation rate
KOOS: Knee injury and Osteoarthritis Outcome Score
MRI: Magnetic resonance imaging
PMN: Polymorphonuclear neutrophil
ROM: Range of motion
SSI: Surgical site infections
WBC: White blood cell
